# Extending the high-throughput toxicokinetics R package to include brain and adipose compartments

**DOI:** 10.3389/fphar.2026.1895108

**Published:** 2026-10-28

**Authors:** Aswani Unnikrishnan, Anna Kreutz, Xiaoqing Chang, James P. Sluka, Dingsheng Li, Matthew W. Linakis, John F. Wambaugh, Nicole Kleinstreuer, Helena T. Hogberg

**Affiliations:** 1 Applied Research Associates, Inc., Raleigh, NC, United States; 2 Inotiv, Research Triangle Park, NC, United States; 3 Biocomplexity Institute, Intelligent Systems Engineering, Indiana University, Bloomington, IN, United States; 4 School of Public Health, University of Nevada Reno, Reno, NV, United States; 5 Ramboll Americas Engineering Solutions, Inc., Research Triangle Park, NC, United States; 6 Chemical Insights Research Institute, UL Research Institutes, Research Triangle Park, NC, United States; 7 National Toxicology Program Interagency Center for the Evaluation of Alternative Toxicological Methods, Division of Translational Toxicology, National Institute of Environmental Health Sciences, Research Triangle Park, NC, United States

**Keywords:** adipose, blood-brain barrier, brain, high-throughput, httk, physiologically based toxicokinetic

## Abstract

Chemical risk assessment has traditionally been performed based on *in vivo* studies. These studies have limited throughput, consequently leading to limited toxicity information for the majority of chemicals in industrial and commercial use. In addition, the translation from animal to human effects can be challenging. To increase human-relevant toxicity knowledge, high-throughput *in vitro* approaches have been developed. However, one challenge in employing these assays for hazard and risk assessment is the translation of *in vitro* bioactive concentrations to *in vivo* doses and internal tissue concentrations. To improve decision-making in chemical risk assessment, as well as drug discovery for humans, the U.S. Environmental Protection Agency has developed a simple physiologically based toxicokinetic (PBTK) model as part of the High-throughput Toxicokinetics open-source R package *(httk*). In this study, the PBTK model was extended to include compartments for the brain and adipose tissues. The model incorporates a blood–brain barrier to control chemical penetration and disposition, which is essential for predicting neurotoxicity. The integration of an adipose tissue compartment further enables the prediction of bioaccumulation, particularly for lipophilic chemicals. The model’s performance was evaluated by comparing predicted chemical concentrations against experimental data from both oral and intravenous routes. This PBTK model extends the applicability of *httk* to allow for assessing potential neurotoxicity and chronic exposure to lipophilic chemicals and better evaluate chemical pharmacokinetics. Moreover, this PBTK model can act as a tool for optimizing dosages and other study parameters to reduce and refine the design of *in vivo* studies.

## 1 Introduction

For the tens of thousands of chemicals in routine industrial and commercial use, only a small fraction has been studied in terms of their toxicity (Judson et al., 2009). While chemical risk assessment has traditionally been based on *in vivo* studies, to adequately fill this information gap it has become increasingly clear that more scalable approaches are needed for risk assessment (Kavlock et al., 2018). *In vitro* and *in silico* tools have been developed to support more rapid risk assessment (Brescia et al., 2023). Many of these methods replicate human biological processes and can perform as well as or better than conventional animal models. However, an inherent challenge in applying data from high-throughput *in vitro* assays to human health risk assessment is relating *in vitro* activity concentrations to human *in vivo* doses (Rotroff et al., 2010). *In vitro* to *in vivo* extrapolation (IVIVE) enables relating an *in vitro* point-of-departure to an external dose required to achieve internal concentrations equivalent to concentrations at which *in vitro* bioactivity was observed (Wetmore, 2015).

Physiologically based toxicokinetic (PBTK) models facilitate IVIVE by providing predictions of concentration-time profiles of chemicals in plasma and individual tissues based on physiological processes. PBTK models can simulate this relationship and are thus central to the application of *in vitro* concentration-response data to risk assessment (Breen et al., 2021; Kreutz et al., 2026). PBTK models use mathematical equations consisting of physiological and chemical-specific parameters representing the absorption, distribution, metabolism, and excretion (ADME) of chemicals in the body, and can be used to predict chemical distribution in different tissue compartments under various dosing scenarios. PBTK models can be developed for a wide range of chemicals through the use of a generic model structure customized with chemical-specific parameter values.

Recognizing the importance of PBTK models to inform and accelerate risk assessment, the U.S. Environmental Protection Agency (EPA) has developed a suite of generic (chemical-independent) PBTK models as part of the open-source High-throughput Toxicokinetics R package *(httk*). Depending upon the model selected, the *httk* package allows researchers to estimate ADME of chemicals following oral, intravenous, or inhalation exposures under perfusion-limited chemical distribution. Using either internal or user-provided physicochemical and ADME properties, *httk* can provide predictions of chemical disposition throughout the body for multiple species. New functionalities and continuous improvement integrated over the last 8 years allow *httk* to perform high-throughput batched assessments for various applications (Pearce R. G. et al., 2017; Pearce et al., 2017 R; Wambaugh et al., 2019; Linakis et al., 2020; Wegner et al., 2020; Breen et al., 2021; Breen et al., 2022; Kapraun et al., 2022).

Two key body compartments not explicitly included in any of the current *httk* PBTK models are adipose (fat) and brain. These tissues are “lumped” with several others into a single “rest of body” compartment. This is due in part to the challenges in estimating chemical partitioning into these compartments. Indeed, a comparison of predictions from models using both the original (Schmitt, 2008) and the modified Schmitt approach to estimate tissue partitioning *in vivo* showed brain and adipose tissue to be amongst the most poorly predicted tissues (Pearce R. et al., 2017). However, chemical distribution into adipose and brain are key considerations for risk assessment.

Many industrial pollutants, particularly persistent organic pollutants like polychlorinated biphenyls and dioxins, are highly lipophilic and are known to accumulate in adipose tissue (Jandacek and Tso, 2001; Jackson et al., 2017). Such lipophilic organic chemicals often undergo slow environmental degradation and are typically metabolized slowly by the liver, eventually accumulating in adipose tissue (Jandacek and Tso, 2001). Deposition into body fat can lead to very long clearance times for these chemicals, which are typically not released until lipolysis, a process that can occur during weight loss and exercise (Jandacek and Tso, 2001; 2024). The accumulation of persistent organic pollutants in adipose tissue has been implicated in the development of obesity and metabolic diseases. The increased concentrations of persistent organic pollutants are also associated with an increased incidence of cardiovascular disease and cancer (Zeliger, 2013). The ability to predict chemical concentrations in adipose tissue is therefore critical from a public health perspective, particularly as plasma levels correlate poorly with adipose tissue concentrations (Jandacek and Tso, 2001).

Another important compartment lacking in the current generic PBTK models within *httk* is the brain, limiting the model’s applicability for assessments of neurotoxicity. As with adipose tissue, estimating brain concentrations is complicated by the high fat content of this tissue (Chang et al., 2009). In addition, the presence of the blood–brain barrier (BBB) increases the complexity of predicting chemical distribution into the brain. In general, the BBB is far less permissive of the transfer of chemicals than the simple endothelial layer that surrounds other tissues. The BBB consists of three major cell types-endothelial cells, pericytes, and astrocytes - which interact to form a barrier limiting transfer of chemicals from the blood into the brain (Ballabh et al., 2004; Kadry et al., 2020). Tight junctions present on the BBB’s endothelial cells particularly limit chemical distribution into the brain, allowing only small molecules and gases capable of passive diffusion across cell membranes to enter freely (Sweeney et al., 2019). Estimating brain concentrations provides a more biologically relevant dose metric for assessing central nervous system effects because it accounts for BBB transport and tissue distribution processes that determine chemical concentrations at sites of neurotoxicity or pharmacological action. In contrast, plasma concentrations alone cannot distinguish chemicals with similar systemic exposure but markedly different brain penetration, potentially leading to inaccurate predictions of central nervous system exposure and associated effects.

Multiple *in vitro* methods have been developed to study chemical passage across the BBB. These range from relatively simple membrane permeability assays simulating passive transport, such as the parallel artificial membrane assay (PAMPA), to complex tri-culture models (Xu et al., 2026) that can capture more comprehensive active transport mechanisms. Effective permeability (P_e_) values from these *in vitro* systems have been shown to correlate with membrane permeability (Tam et al., 2010) and can be used in PBTK models to predict brain concentrations of chemicals.

The *httk* package is open-source and extensible, and new models can be added using the GNU simulation software MCSim (Bois, 2009) and the R programming language (Davidson-Fritz et al., 2025). Using this extensibility, we have incorporated adipose and brain compartments into *httk* for humans and rats*,* as shown in Figure 1, providing a broadly applicable extension intended to support large-scale toxicity screening rather than any specific chemical category. The adipose compartment was added using the modified Schmitt approach based on simple perfusion for estimating tissue partitioning. For the brain, we mimicked passive passage across the BBB through PAMPA P_e_ values available for a broad set of chemicals. Extending the standard *httk* PBTK model to include explicit adipose and brain compartments will facilitate broader applicability of this open-source tool for risk assessment.

**FIGURE 1:**
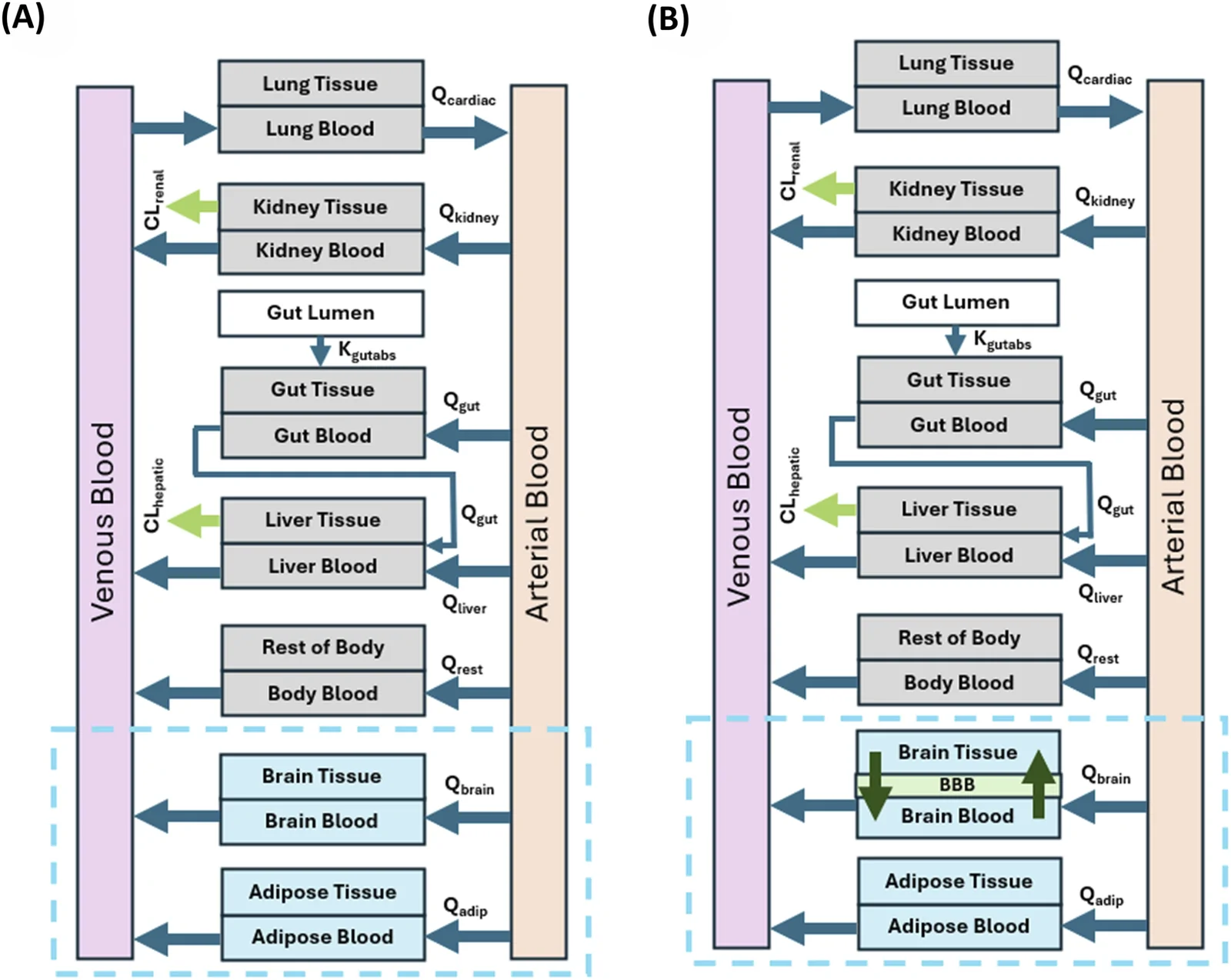
Layout of the extended *httk* model including adipose and blood compartments. The standard *httk* compartments are shown in grey. The newly added compartments, shown in blue, are for brain and adipose. **(A)** Perfusion-limited model. **(B)** Variation of the model incorporating a diffusion-limited compartment for the brain incorporating BBB permeability (dark green arrows). Both models show two clearance pathways (light green arrows) via the kidneys (CL_renal_; renal clearance) and the liver (CL_hepatic_; hepatic clearance), as well as cardiac output (Q_cardiac_), organ specific blood flow rates (Q_kidney_, Q_gut_, Q_liver_, etc.) and the rate constant of gut absorption (K_gutabs_).

## 2 Materials and methods

PBTK modeling was conducted using the *httk* R package (version 2.2.2). The *httk* package includes standard physiological parameters such as tissue volumes and blood flow rates for multiple species. The package also includes physicochemical and toxicokinetic data for thousands of chemicals. These include experimental data from both *in vitro* and *in vivo* assays, where available, or predictions of these values derived from quantitative structure–activity relationship models such as the Open (Quantitative) Structure–activity/property Relationship App (OPERA; Mansouri et al., 2018). Tissue partitioning in *httk* was estimated using a modified Schmitt approach (Pearce R. et al., 2017; Breen et al., 2021; 2022). The default *httk* physiological parameters were used for all simulations unless otherwise specified in Supplementary Data Tables 1.1, 1.2. For chemical-specific parameters, experimentally measured values were used when available, followed by predicted values when measured values were not reported. In cases where *httk* provided an internal default (e.g., for fraction of chemical unbound in plasma), that value was used for chemicals lacking either a measured or a predicted value. The default body weight was used; for humans, this was 70 kg, and 0.25 kg for rat. Standard allometric scaling was applied to represent individuals with different body weights. Most tissue compartments in our modeling package operated under a perfusion-limited assumption, where blood flow was the primary determinant of the rate at which a chemical is taken up into a tissue. We initially used the perfusion-limited assumption to model the brain compartment (Figure 1A) and then further refined the model to include a BBB component, which used a specialized diffusion-limited assumption (Figure 1B). This modification of the brain compartment incorporated specific barrier kinetics, which improved upon the perfusion-limited assumption by more accurately representing the restricted transfer of chemicals across the BBB. All other modeled compartments retained the perfusion-limited assumption. Hence, in our modeling framework, adipose tissue is represented using a perfusion-limited structure, reflecting that chemical uptake and release are predominately governed by blood flow. This assumption is consistent with the physiological role of adipose as a highly vascularized storage compartment where passive distribution is the major driver of chemical exchange. In contrast, the brain compartment incorporates an explicit permeability term to represent the BBB. The BBB imposes an additional resistance to passive chemical movement not present in most other tissues, necessitating a diffusion-limited approach in which transport across the endothelial interface is restricted by the estimated permeability coefficient. The model code, which is available through the Zenodo repository, uses the specialized diffusion-limited assumption for the BBB, with perfusion-limited distribution for the remaining tissue compartments.

### 2.1 Software and tools used

Our modification of the *httk* package to add brain and adipose compartments was based on the methods of Davidson-Fritz et al. (2025). The model’s base framework used existing *httk* functions and was developed within the R statistical computing environment (version 4.3.0 and later). We defined the PBTK model structure using the GNU MCSim (https://github.com/HSL/MCSim) simulation package (version 6.1.0), which generated C code via the “mod” command-line utility. The MCSim mod command-line utility compiled the model’s ordinary differential equations (ODEs) specification file into optimized C code, which was then used for fast numerical simulation within the revised *httk* package. This step translated the PBTK model structure into executable code that was integrated into the package’s src directory for use by the model solving functions. This code was then compiled and integrated into the new R package.

The workflow for updating *httk* is shown in Figure 2. The *httk* adipose-brain extension software package (httk_2.2.2.tar.gz), together with the accompanying output vignette (output_vignette.Rmd) and its Word output document, is available in the Zenodo repository (https://doi.org/10.5281/zenodo.22149600). In this repository, we provide all code and scripts required to reproduce the figures and validation results for the diffusion-limited brain compartment model, including explicit BBB permeability and the associated perfusion-limited adipose compartment. The repository also includes MassBalanceCheck.Rmd and its corresponding Word output document, which demonstrate a mass-balance check using bisphenol A as an example. An earlier version of the model used during initial model exploration and development, which incorporated a perfusion-limited brain compartment, was not included in the final package or the vignette. The vignette and all reproducible code provided in the repository correspond to the finalized diffusion-limited brain compartment model and its paired adipose compartment workflow. Because the perfusion-limited brain compartment model produces output using the same scripting structure, providing a separate vignette for that preliminary version would be redundant.

**FIGURE 2:**
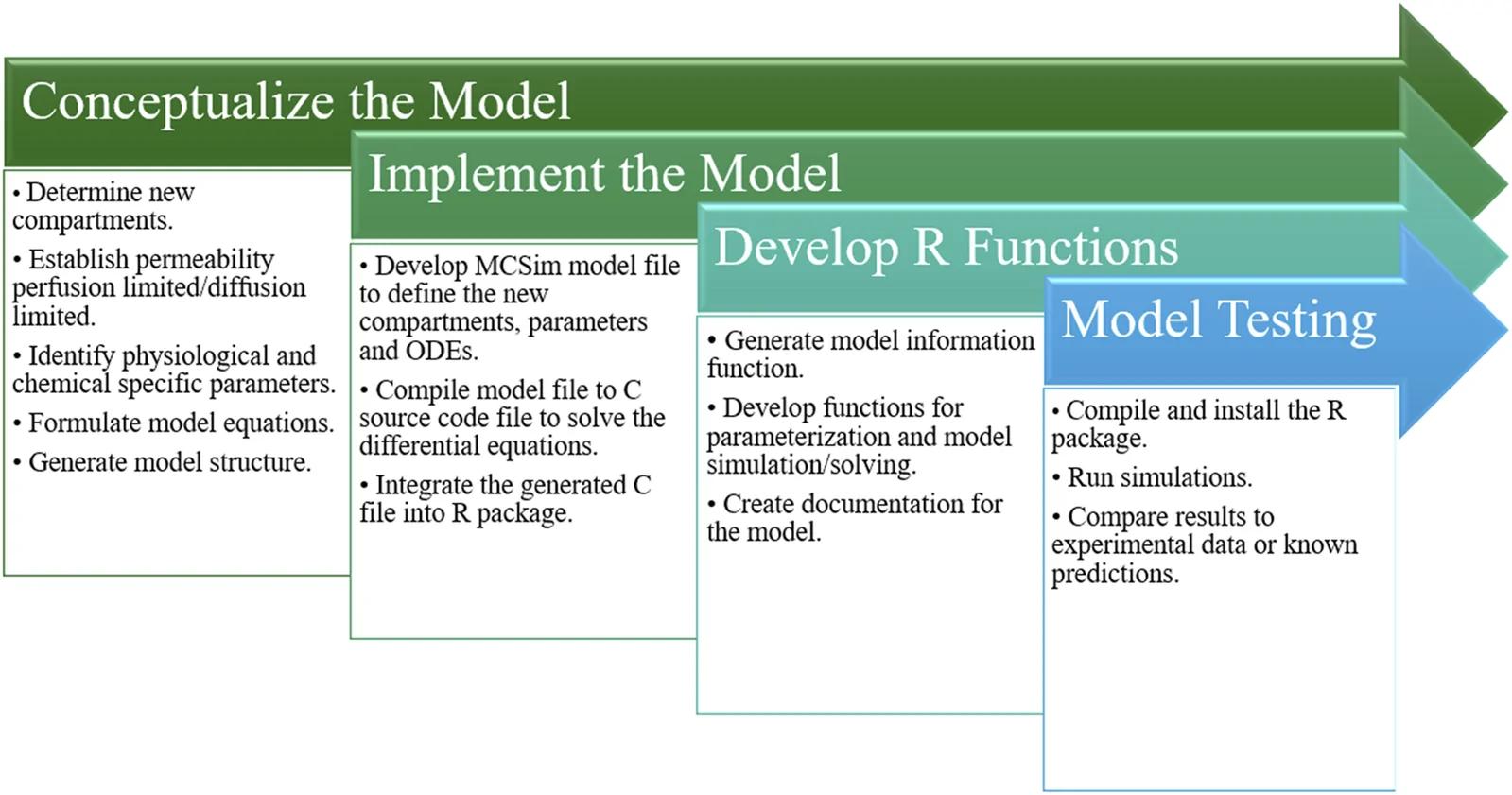
Workflow for developing new *httk* models, based on Davidson-Fritz et al. (2025).

For more information on the methodology behind adding models to *httk* and the interplay between different software, please refer to Davidson-Fritz et al. (2025).

### 2.2 Model development

The new model started with the most basic PBTK model within *httk*–which is simply called “pbtk” (Pearce R. G. et al., 2017). To separate the brain and adipose tissues from the original *httk* “Rest-of-Body” compartment and assign each tissue its own explicit compartment, we defined sets of ordinary differential equations representing these two compartments. Their corresponding tissue volumes and blood-flow fractions were reassigned and subtracted from the aggregated Rest-of-Body parameters to maintain physiological consistency, including preservation of total body weight, cardiac output allocation, and overall mass balance. A mass-balance check was implemented in the MassBalanceCheck.Rmd file to evaluate mass conservation in the updated PBTK model. At each time point, the total amount of chemicals in the blood and all tissue compartments, together with the amounts eliminated or metabolized, were calculated and compared with the administered dose. The relative mass-balance error was calculated as the difference between the total accounted mass and the administered dose, normalized to the administered dose. Using bisphenol A as an example, the model demonstrated mass balance with a relative error of <5 × 10^−4^ at a dose of 1 mg/kg/day (Supplementary Figure S10). The differential equations were then translated into C code using the MCSim mod utility, which compiles model specifications into optimized executable code for efficient numerical simulation. The resulting C implementation, modelpbtk_adipose_complexbrain.c, was added to the *httk* package’s source directory and integrated with the updated R functions that support parameterization, model information, and simulation process.

To implement the extended brain and adipose compartments, we modified several components of the standard *httk* framework. The model structure, parameterization functions, and solver routines were extended by updating key files within the package, including get_physchem_param.R, parameterize_pbtk_adipose_complexbrain.R, modelinfo_pbtk_adipose_complexbrain.R, solve_pbtk_adipose_complexbrain.R. The modified R package was then compiled to include the modified models (Figure 2). Users can apply the “solve_pbtk_adipose_complexbrain” function to call this model, which is consistent with the existing *httk* models. These modifications followed the established workflow described by Davidson-Fritz et al. (2025), which outlines how compartment structures and simulation functions are added within the *httk* architecture. In addition to the modified model files, the adipose-brain extension makes use of the chem.physical_and_*invitro*.data and physiology.data resources already included within the standard *httk* package, which provide curated chemical-specific properties and species-specific physiological parameters. These datasets were supplemented with updated entries for P_e_ and brain surface area to support the added brain and adipose compartments.

Tissue-specific partition coefficients are predicted using a modified version of the method of Schmitt (2008) as implemented in the function “predict_partitioning_schmitt”. That method describes chemical partitioning as a function of the octanol:water partition coefficient, chemical ionization, plasma protein binding, and the fractional composition of each tissue (for example, water vs. phospholipid). Pearce R. et al. (2017) empirically calibrated the predictions for each tissue using a dataset of measured partition coefficients, and these calibrations are applied here. This model will be submitted via the EPA GitHub repository (https://github.com/USEPA/CompTox-ExpoCast-httk) for consideration in a new version.

#### 2.2.1 Adipose compartment

Adipose tissue was modeled as a simple, perfusion-limited compartment, accounting for its role as a storage site for lipophilic chemicals. The chemical distribution to the adipose compartment is modeled using the following equations:

$$\frac{{dA}_{adipose}}{dt}=Q_{adipose}\left(C_{art}-\frac{C_{adipose}\cdot R_{b2p}}{K_{adipose2pu}{.f}_{up}}\right)$$ (1)

$$Q_{adipose}=Q_{cardiac}\cdot Q_{adiposef}$$

$$V_{adipose}=V_{adiposec}\cdot BW$$

In Equation 1, A_adipose_ refers to the total amount of a chemical present in the adipose compartment, while Q_adipose_ represents the blood flow delivered to adipose tissue, calculated as a fixed fraction of the total cardiac output (Q_cardiac_ × Q_adiposef_). C_art_ is the concentration of chemicals in arterial blood, and C_adipose_ is the concentration of the chemical in adipose tissue. The unbound fraction in plasma, denoted as f_up_, indicates the portion of the chemical that is not bound to plasma proteins and is thus available for distribution into tissues. The blood-to-plasma concentration ratio (R_b2p_) relates the concentration of the chemical in whole blood to that in the plasma. Additional parameters include body weight (BW) and the total adipose tissue volume (V_adipose_). The V_adipose_ parameter defines the size of the adipose compartment and its capacity for chemical distribution and is computed as the product of BW and the adipose tissue volume fraction (V_adiposec_). Q_cardiac_ provides the overall cardiac output, and Q_adiposef_ specifies the fractional allocation of that output directed to adipose tissue. The adipose-to-plasma partition coefficient (K_adipose2pu_) is a critical parameter representing the ratio of the chemical’s concentration in adipose tissue relative to its unbound concentration in plasma. This parameter is derived using the modified Schmitt tissue-partitioning approach (Schmitt, 2008), which estimates the ratio of a chemical’s concentration in adipose tissue to its unbound concentration in plasma based on chemical-specific properties such as octanol:water partition coefficient (logP), acid dissociation constant, membrane affinity, plasma protein binding, adipose-specific tissue composition, and pH. K_adipose2pu_ is calculated internally from the chemical’s physicochemical inputs using the same general Schmitt approach applied across all tissues.

#### 2.2.2 Brain compartment

Brain was modeled as a complex compartment to represent the diffusion-limited transport of chemicals into the brain through the tight junctions of the BBB. This model enables distinguishing between the brain capillary blood and brain tissue and is limited to passive permeability; it does not capture active transport. Consequently, the model may not adequately predict brain concentrations for chemicals that are substrates of active BBB transporters, e.g., P-glycoprotein. For such chemicals, observed brain exposure may differ substantially from predictions based solely on passive permeability and tissue partitioning. Chemicals identified as having substantial active transporter involvement were therefore considered outside the applicability domain of the current brain model and were excluded from model evaluation.

In a diffusion-limited brain compartment model, the vascular surface area is needed to calculate the transfer rate across the BBB given that the effective permeability is expressed as a rate of cm/s. Reported literature values for the surface area of BBB microvessels in humans range between 12 and 25 m^2^ (Nag and David, 2005; Abbott et al., 2010; Wong et al., 2013; Mehta et al., 2024). We therefore used the average of these: 17.5 m^2^. For rats, estimates reported for the surface area of the rat BBB range between 260 and 300 cm^2^. We opted to use the same value as used by Mehta et al. (2024), as it falls in this range and was explicitly provided in an open-source model.

Transfer of a chemical to the brain tissue was modeled using the following equations:

$${\frac{d}{dt}A_{brain,b}=Q}_{brain}.\;\left(C_{art}-C_{brain,b}\right)-K_{pbbb}.\;Q_{brain}.\;\left(C_{brain,b}-\frac{C_{brain}.\;R_{b2p}\;}{K_{brain2pu}.\;f_{uP}}\right)$$ (2)

$$\frac{d}{dt}A_{brain}=K_{pbbb}.Q_{brain}.\left(C_{brain,b}-\frac{C_{brain}{.R}_{b2p}}{K_{brain2pu}.f_{up}}\right)$$ (3)

In these equations, A_brain,b_ is the amount of chemical in the capillary blood of the brain; A_brain_ is the amount of chemical present in the brain tissue compartment; Q_brain_ is the blood flow through the brain; C_art_ is the concentration of chemical in arterial blood; C_brain,b_ is the concentration of chemical in the capillary blood of the brain; R_b2p_ is the blood-to-plasma concentration ratio, which relates the concentration of the chemical in whole blood to that in the plasma; K_brain2pu_ is the tissue-to-unbound plasma partition coefficient; f_up_ is fraction of chemical unbound in plasma; C_brain_ is the chemical concentration in brain tissue. K_pbbb_ is the permeability coefficient between capillary blood and brain (representing the BBB) and is the key parameter that differentiates the brain from other compartments. This parameter is the one that is most difficult to measure or predict. We used PAMPA values to estimate this parameter; we therefore inherit any uncertainty associated with those values or their applicability to predictions made for the *in vivo* situation.

Equation 2 describes the change in the amount of chemical in the capillary blood of the brain, with chemical distribution from capillary blood to the brain tissue represented as a diffusion-limited process. The movement of the chemical from capillary blood to the cellular matrix of the brain tissue is proportional to the product of permeability and surface area. Assuming the surface area is proportional to the regional blood flow, this can be expressed as a proportion of the blood flow Q_brain_ due to the BBB effect represented by K_pbbb_.

In Equations 2, 3, K_pbbb_ is a unitless ratio essentially limiting the total blood flow for the brain, described by Equation 4. The parameter P_e_, effective permeability, is measured in units of cm/s (often expressed in quantities of 10^–6^). The surface area of the brain, Area_brain_ (cm^2^), is multiplied by P_e_ to yield a product with a unit of cm^3^/s (or mL/s). This represents the reduction of blood flow as a result of the permeability-limited process. The ratio of that product and Q_brain_ will be the K_pbbb_, thus calculated as:

$$K_{pbbb}=\max\left(\frac{P_{e}\;.\;{Area}_{brain}}{Qbrain},1\right)$$ (4)

(P_e_) has a unit of cm/s.

Dimensional analysis, units added to each term:

$$K_{pbbb}=\frac{P_{e}\frac{cm}{s}\cdot {Area}_{brain}{cm}^{2}}{Qbrain\frac{{cm}^{3}}{s}}$$

Term names removed leaving only units:

$$K_{pbbb}=\frac{\frac{cm}{s}\cdot {cm}^{2}}{\frac{{cm}^{3}}{s}}=unitless$$

Since K_pbbb_ is effectively a modifier representing how the BBB passively limits blood flow into the brain compartment, it cannot exceed the value of 1. A value of greater than one would imply that the BBB could increase blood flow to the brain, which is not possible when the model only considers passive transport. The BBB permeability surface area product was based on measured assay data or predictions from a separate *in silico* model (Illa et al., 2025). This model was developed to predict BBB permeability using data from a PAMPA for brain membranes (PAMPA-BBB), aiming to improve the prediction of *in vivo* brain exposure from *in vitro* tests. The model, trained on 106 chemicals, showed a predictive accuracy R^2^ of 0.71 (Illa et al., 2025). Our approach prioritized the use of experimentally measured P_e_ values, which were gathered for 113 chemicals from a multitude of literature sources. Where direct measurements were not available, we used predicted P_e_ values from the Illa et al. (2025) model. In cases where no measured or predicted P_e_ value was available, we excluded that chemical from model evaluation. Consequently, the reliability of the predicted brain concentrations is inherently dependent on the applicability of the underlying PAMPA-BBB measurements used in the model. Any experimental variability or systematic bias in these *in vitro* data will propagate into the predicted values and, ultimately, into estimates of brain exposure. Predicted brain concentrations represent total brain concentrations.

### 2.3 Model evaluation

Concentration versus time (CvT) data were gathered for plasma, adipose, and brain tissue to support evaluation of the adipose-brain PBTK model. CvT data were limited to human or rat species and oral or intravenous routes of administration. CvT data were gathered through a literature search, the EPA’s CvT database (Sayre et al., 2020), and the National Institute of Environmental Health Sciences’ Chemical Effects in Biological Systems (CEBS) database (https://cebs.niehs.nih.gov/cebs/).

The literature search was conducted using PubMed with the terms “concentration AND time” and “brain,” “adipose,” or “fat,” along with “rat” or “human”. This literature was reviewed for CvT data. In instances where CvT values were only available in plots rather than tabular form, the Webplot digitizer tool (https://automeris.io/WebPlotDigitizer/) was used to extract numerical values by calibrating the plots, manually selecting the plotted points, and downloading the corresponding data. We verified that the digitized data were an accurate representation of the published graphs by comparing the digitized data with the original graph.

Data from CEBS were extracted from a download of all available toxicokinetic data on 1 July 2024 (Lea et al., 2017). Data were gathered from the EPA’s CvT database for adipose, fat, brain, and plasma. Regardless of the extraction method, data were reviewed as part of the model evaluation process. Not all of the gathered data were used for model evaluation based on limitations of our model and applicability domain considerations, as is documented in Supplementary Data Table 1.8. Several chemicals were initially excluded from model evaluation because they lacked a Chemical Abstracts Service Registry Number in *httk*. For chemicals lacking values for fraction of chemical unbound in plasma and intrinsic clearance, OPERA predictions for these parameters were obtained from the National Institutes of Health Integrated Chemical Environment (https://ice.ntp.niehs.nih.gov/), with appropriate consideration of applicability domain. Predictions that fell outside OPERA’s applicability domain were not used, and the chemical was removed from the final evaluation set. Active transport is known to play a critical role in chemical brain distribution and would generally cause chemical concentrations to be overpredicted in brain, due to the fact that the major transporters on the BBB, P-glycoprotein and BCRP, are efflux transporters. Therefore, chemicals that were identified by Yamamoto et al. (2017) as transporter substrates through the co-administration of transporter inhibitors were also dropped from the final evaluation set. Since the publicly available release of *httk* includes exclusions for per- and polyfluoroalkyl substances (PFAS), we chose to evaluate model performance both with and without PFAS.

PBTK modeling was performed on identified chemicals that had experimental CvT data obtained from literature or database sources. Simulations generated using our model used the same dosing scenarios (dose, days of dosing, doses per day, and route of exposure) as were used to generate the corresponding experimental data used as model inputs for both single and repeated dosing schedules. Gavage and other routes of oral administration were modeled via the oral route by simply using the original dose from the publication and setting IV to “False,” which then defaults to the oral route for the PBTK model. For chemicals lacking sufficient data to perform PBTK modeling, toxicokinetic data, e.g., fraction unbound in plasma and hepatic clearance, were gathered from the EPA CompTox Chemicals Dashboard (https://comptox.epa.gov/dashboard/), taking experimental over predicted values where available.

Our updated *httk* model including the brain and adipose compartments was used to simulate chemical CvT profiles for various scenarios, including different exposure routes (oral, intravenous) and chemical properties like lipophilicity. The simulated CvT profiles for brain and adipose tissue were compared against a curated set of independent experimental data obtained from the literature as described above. Model performance was evaluated based on the fold difference in maximum concentration (C_max_) between predicted and experimental values for within-tissue concentration on a per dosing scenario and per chemical basis. To characterize overall predictive behavior for the adipose and brain compartments, we calculated standard diagnostic metrics, including the Pearson correlation coefficient of determination (R^2^) and the root mean square error (RMSE). R^2^ describes the proportion of variance in the observed log_10_ C_max_ values explained by the model, whereas RMSE (log_10_) quantifies the average magnitude of prediction error on the log scale. RMSE was calculated using log-transformed C_max_ values, not the original concentration values. Specifically, observed and predicted C_max_ values were first transformed using log_10_. A linear regression was then fit to log_10_(predicted) versus log_10_(observed), and RMSE was computed from the residuals of that regression, representing the average deviation of the log-transformed predictions from the fitted line. This makes RMSE a measure of error on the log_10_ scale rather than in μM units.

In addition, mean absolute log error (MALE) was used to summarize the average absolute deviation between predicted and observed log_10_ C_max_, and mean fold error (MFE) and geometric mean fold error (GMFE) were used to express typical multiplicative prediction error on the original concentration scale. We also report the percentage of predictions falling within 2-fold, 5-fold, and 10-fold of observed values to provide an intuitive measure of agreement. All performance metrics described above, including slope, R^2^, RMSE (log_10_), MALE, MFE, GMFE, and the percentages of predictions within 2-, 5-, and 10-fold of observed values, were calculated for the brain compartment both with and without PFAS.

Chemicals with predictions that differed from experimental data by more than 10-fold were reviewed for additional properties that might impact the predictive performance of our model and are discussed in the Results and Discussion.

## 3 Results

### 3.1 Model demonstration

To illustrate expected behavior of the perfusion-limited and diffusion-limited brain compartment models, we generated CvT profiles for both the adipose and brain compartments across several chemicals. These examples show the model producing realistic compartmental dynamics under the dosing scenarios reported in the source studies (routes and dose intervals as specified). Figure 3 compares observed and predicted acetaminophen CvT profiles for adipose tissue using the diffusion-limited brain compartment model (Figure 3A) and brain under perfusion-limited (Figure 3B) and diffusion-limited brain compartment (Figure 3C) model assumptions. Additional demonstration plots for benzo(a)pyrene, dieldrin, and trichloroethylene are provided in Supplementary Figures S1–S6, with supporting data provided in Supplementary Data Table (C_max_ values in sheet 1.7; log10 C_max_ values for both compartments in sheets 1.5 and 1.6). The figures as a group illustrate how time-course alignment for the diffusion-limited brain compartment model varies across chemicals. Poor alignment of the curves may indicate cases for which the model likely is not capturing certain chemical-specific kinetics such as tissue binding or active transport, which can cause the overall shape of the time-course to be off even when the C_max_ values are close.

**FIGURE 3:**
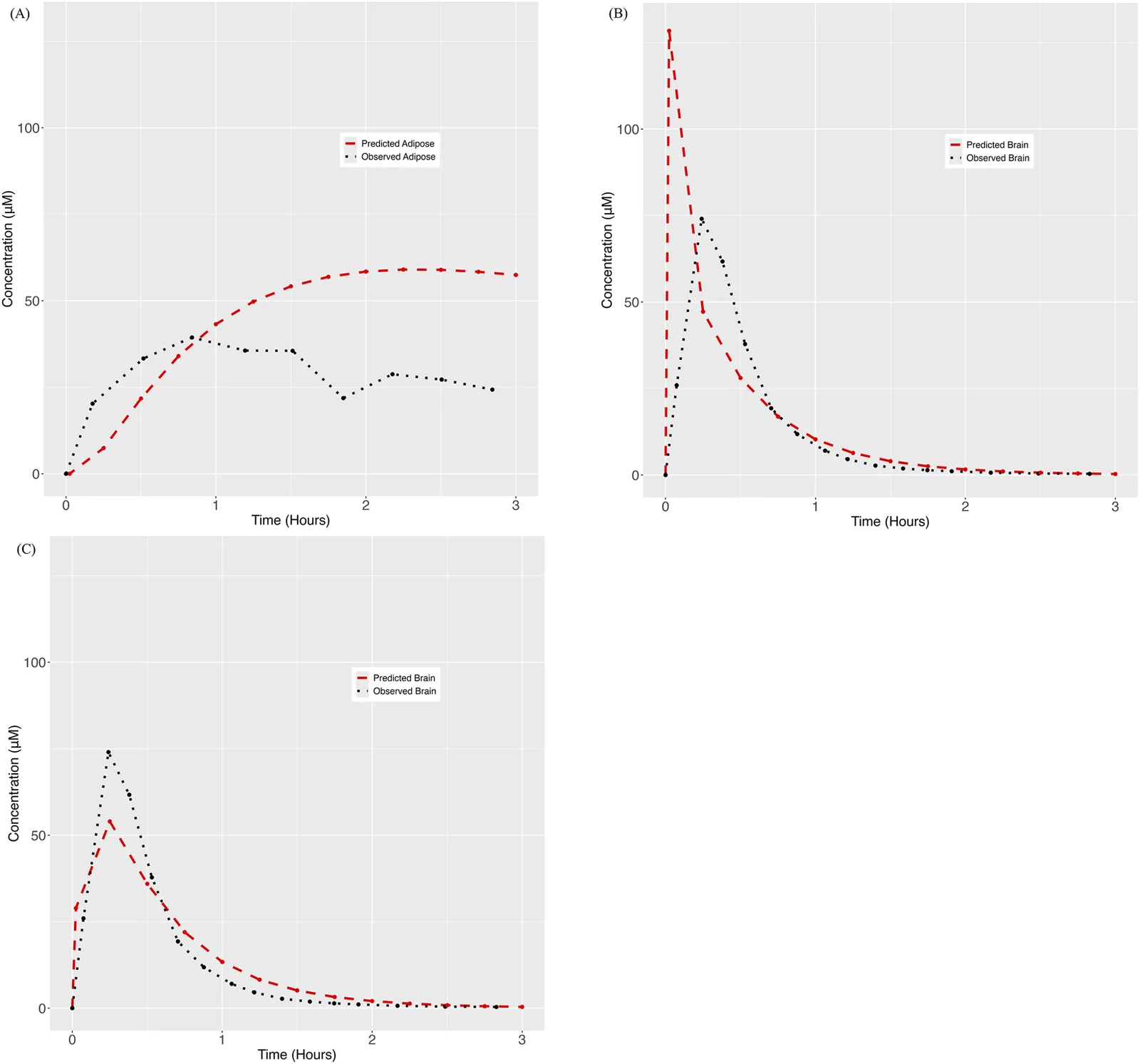
**(A)** acetaminophen experimental CvT data for adipose tissue (dotted black line) compared to modified *httk* predictions (dashed red lines) for the adipose compartment in the diffusion-limited brain compartment model. Input parameters: K_pbbb_ = 0.14, daily dose = 14.28 mg/kg, days of dosing = 1, doses per day = 1, species = human, route = oral. **(B,C)** acetaminophen experimental CvT data for brain (dotted black lines) compared to both modified *httk* predictions (dashed red lines) from the **(B)** perfusion-limited model and **(C)** diffusion-limited brain compartment model. Input parameters: daily dose = 16 mg/kg, days of dosing = 1, doses per day = 1, species = rat, route = IV. The diffusion-limited brain compartment model **(C)** assumed a K_pbbb_ = 0.12.

### 3.2 Model evaluation

*In vivo* experimental CvT data were available for eight chemicals for adipose tissue and 41 chemicals for the brain compartment. Several of the chemicals with available brain data were removed due to various parameterization or applicability domain limitations, including consideration of the chemical being a transporter substrate, which would result in the chemical being overestimated by our model due to the efflux transporters on the BBB (Supplementary Data Table 1.8). After this curation, 22 chemicals were left for evaluation.

The predictive capability of the diffusion-limited brain compartment model (Figure 1B) was evaluated by comparing its CvT estimates to these experimental data. The species and dosing regimens used for these estimates were derived from the experimental studies and are detailed in Supplementary Data Tables 1.3, 1.4. Each distinct dosing regimen (e.g., route, dose interval) was treated as an independent observation. C_max_ values were derived to provide a quantitative metric for model evaluation, and R^2^ and RMSE were calculated to evaluate overall model performance.

Model predictions of C_max_ values were mostly within 5-fold of experimental measurements for both adipose and brain compartments. For the adipose compartment, five of the eight chemicals evaluated fell within a 5-fold range of experimental C_max_ values. The adipose performance metrics reflected this pattern, with a slope of 0.94, R^2^ of 0.96 (95% confidence interval [CI]: 0.94–0.98), RMSE (log_10_) of 0.70 (95% CI: 0.57–0.82), MALE of 0.57, MFE of 0.41, GMFE of 3.7, and 31.7%, 70.7%, and 78% of predictions falling within 2-, 5-, and 10-fold of observed values, respectively (Figure 4). For the brain compartment, fold-error results were more variable across chemicals. When all chemicals were included, the MFE was 3.2, with 29%, 56.5%, and 72.6% of predictions falling within 2-, 5-, and 10-fold of observed values, respectively. Corresponding model performance metrics were slope 0.75, R^2^ 0.71 (95% CI: 0.55–0.83), RMSE (log_10_) 0.83 (95% CI: 0.69–0.96), MALE 0.67, and GMFE 4.7 (Figure 5).

**FIGURE 4:**
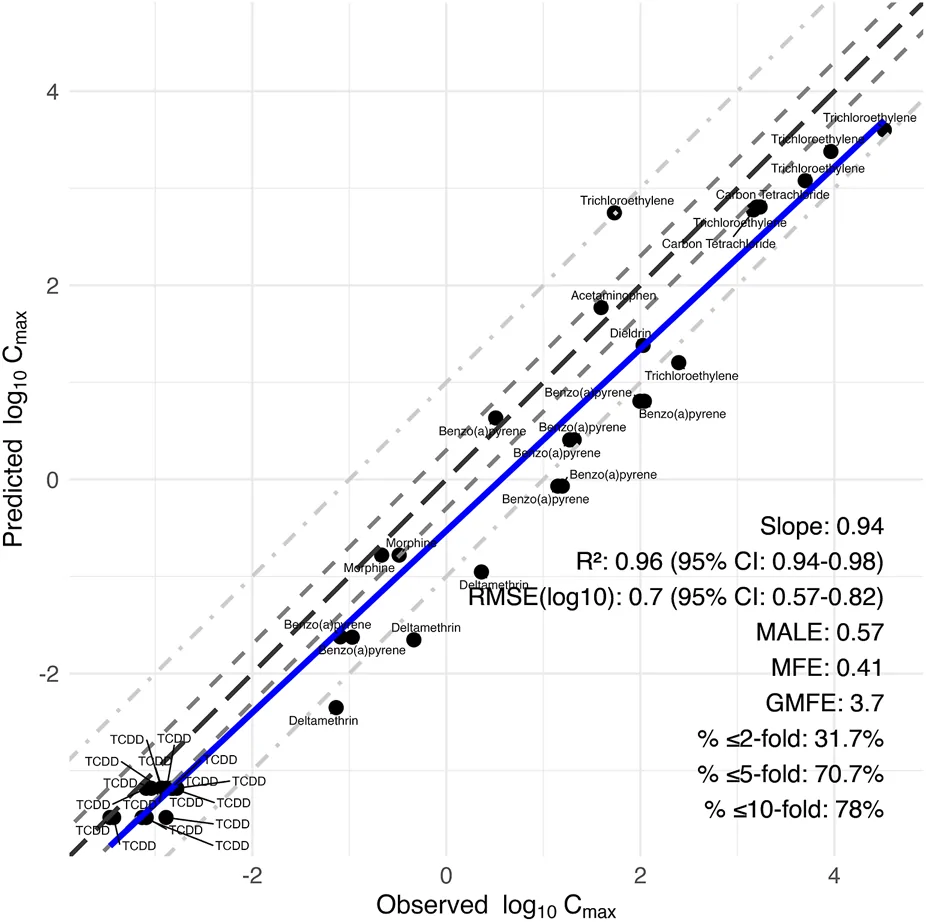
Predicted versus observed log C_max_ values for adipose in the diffusion-limited brain compartment model, shown across eight chemicals and 40 dosing regimens for the respective species. Dark grey long-dashed line is the line of identity, blue solid line represents the regression line, grey short-dashed lines show ±2-fold, and the grey dot-dashed lines show ±10-fold.

**FIGURE 5:**
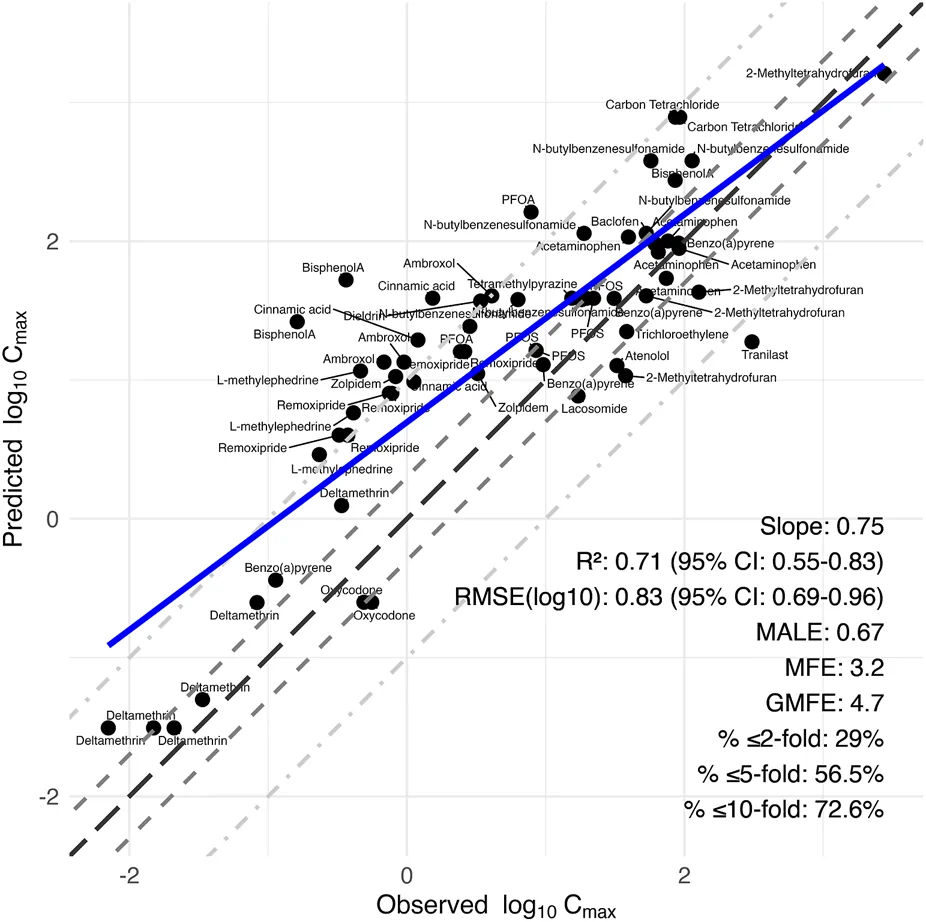
Predicted versus observed log C_max_ values for brain in the diffusion-limited brain compartment model, shown across 22 chemicals and 62 dosing regimens for the respective species. Dark grey long-dashed line is the line of identity, blue solid line represents the regression line, grey short-dashed lines show ±2-fold, and the grey dot-dashed lines show ±10-fold.

Excluding the two PFAS chemicals that remained following our exclusion criteria resulted in minimal changes to performance (Supplementary Figure S9), with resulting slope 0.75, R^2^ 0.72 (95% CI: 0.56–0.83), RMSE (log_10_) 0.84 (95% CI: 0.69–0.99), MALE 0.68, MFE 3.3, GMFE 4.8, and 25%, 55.4%, and 71.4% of predictions within 2-, 5-, and 10-fold. Based on the curated dataset, 12 of the 22 total chemicals included in the brain evaluation fell inside the 5-fold range, while 10 fell within a 3-fold deviation from experimental values (Table 1; Supplementary Data Tables 1.5, 1.6).

**TABLE 1:** Table representing the comparison of model-predicted versus experimental C_max_ values. Mean pred_max: mean predicted C_max_; Mean exp_max: mean experimental C_max_ based on PBTK modeling; GMFE: geometric mean fold error of experimental vs. predicted values.

Compartment	Chemical	Mean pred_max	Mean exp_max	GMFE
Brain	2-Methyltetrahydrofuran	427.52	744.1	2.20
	Acetaminophen	86.79	69.19	1.45
	Ambroxol	22.43	1.91	14.00
	Atenolol	12.71	32.63	2.57
	Baclofen	95.91	61.32	1.56
	Benzo [a]pyrene	37.20	32.91	1.54
	BisphenolA	117.87	28.77	42.19
	Cinnamic acid	22.66	1.29	15.25
	Deltamethrin	0.27	0.08	2.46
	Dieldrin	37.06	3.40	10.90
	Lacosomide	7.68	17.12	2.23
	L-Methylephedrine	6.77	0.37	16.37
	N-Butylbenzenesulfonamide	177.19	44.57	3.91
	Oxycodone	0.25	0.52	2.09
	Perfluorooctanoic acid	93.27	5.35	13.31
	Perfluorooctanesulfonic acid	27.57	14.70	1.89
	Remoxipride	9.35	1.21	9.16
	Carbon tetrachloride	783.10	89.34	8.77
	Tetramethylpyrazine	38.86	15.42	2.52
	Tranilast	18.83	306.78	16.29
	Trichloroethylene	22.33	38.36	1.72
	Zolpidem	10.86	2.03	6.63
Adipose	Benzo[a]pyrene	2.66	31.31	7.74
	Carbon tetrachloride	641.80	1650.13	2.57
	Deltamethrin	0.05	0.95	19.22
	Dieldrin	24.03	106.82	4.45
	Morphine	0.17	0.27	1.60
	Paracetamol	59.01	39.38	1.50
	2,3,7,8-Tetrachlorodibenzodioxin	5.60E-04	1.10E-03	1.89
	Trichloroethylene	1459.21	8119.51	6.11

For acetaminophen, the diffusion-limited brain compartment model predicted a C_max_ of 54.01 µM, which is within approximately 2-fold of the observed brain concentration of 74.03 µM (Figure 3C; Supplementary Data Table 1.4). Similarly, in adipose compartment, the model predicted C_max_ of 59.01 µM for acetaminophen was within 2-fold of the observed value of 39.38 µM (Figure 3A; Supplementary Data Table 1.3).

The quantitative metrics for the adipose compartment in the model with the diffusion-limited brain compartment (Figure 4) were similar to those from the perfusion-limited brain compartment model (Supplementary Figure S7). We also compared the performance of brain compartment in the diffusion-limited brain compartment model (Figure 5) against the earlier perfusion-limited brain compartment model (Supplementary Figure S8) using the same 22 curated chemicals and 62 dosing regimens. The diffusion-limited brain compartment model demonstrated better predictive performance across the quantitative metrics, including improved slope (0.75 vs. 0.69), higher R^2^ (0.71 vs. 0.69), lower RMSE on the log_10_ scale (0.83 vs. 1.00), and reduced mean absolute log error (0.67 vs. 0.85). Fold-error diagnostics likewise favored the diffusion-limited brain compartment model, with MFE and GMFE substantially reduced (MFE 3.2 vs. 8.0; GMFE 4.7 vs. 7.1). Accuracy bands also showed notable gains, with a larger proportion of predictions falling within 2-, 5-, and 10-fold of observed values (29%, 56.5%, and 72.6% for diffusion-limited brain compartment model, compared with 22.6%, 43.5%, and 64.5% for the perfusion-limited brain compartment model).

Together, these results describe the performance of our diffusion-limited brain compartment model for both compartments based on the curated dataset.

## 4 Discussion

This work builds upon the fields of PBTK modeling and high-throughput toxicokinetic modeling by expanding the capabilities of *httk*, a key PBTK model in the field (Geci et al., 2024; Damre and Banerjee, 2025; Spaenig et al., 2026). PBTK models provide a mechanistic, data-driven approach to support science-based decision-making, while reducing the need for animal testing. In high-throughput applications they further support more rapid, cost-effective screening of chemicals. PBTK models have applications in both the drug development arena, such as for dose optimization, and in the regulatory arena, where they can be used in risk-based chemical prioritization and high-throughput exposure and safety evaluations (Damre and Banerjee, 2025). Recognizing the benefits PBTK models can provide, the European Medicines Agency, the U.S. Food and Drug Administration, and the Organisation for Economic Cooperation and Development have issued guidance on the use of PBPK/PBTK modeling for regulatory applications (OECD, 2021; Damre and Banerjee, 2025).

The expansion of *httk* described here provides refined quantitative predictions of chemical distribution in two toxicologically relevant compartments, the brain and adipose tissue, for both humans and rats. By building upon an existing open-source PBTK framework, this new model addresses a notable limitation in high-throughput models where dedicated brain and adipose tissue compartments are often simplified or absent. *httk* is intended to be an extensible environment for model development and distribution (Davidson-Fritz et al., 2025). Other recent expansions of *httk* include PBTK models allowing for a dermal exposure route (Meade et al., 2026) and modifications for per‐ and polyfluoroalkyl substances (PFAS) (Wambaugh et al., 2026).

The importance of including adipose (Albanese et al., 2002; Spaenig et al., 2026) and brain (Ball et al., 2013) tissues as distinct compartments in PBTK models has been previously noted. The inclusion of these specific tissues as distinct compartments supports more comprehensive chemical risk assessment than what is currently available. The predictions for adipose tissue provide insight into the potential for bioaccumulation and long-term exposure to persistent chemicals. The model’s brain compartment explicitly accounts for the BBB, which is a significant permeability barrier for many chemicals. This enables more accurate modeling of chemical distribution into the central nervous system. The resulting chemical CvT profiles in these tissues provide critical internal exposure estimates; brain CvT profiles can inform neurotoxicity assessments, and adipose profiles help in understanding the risks associated with long-term exposure. Species-specific physiological parameters, such as body weight and vascular surface area, are systematically applied within the model to reflect differences between rats and humans, which is particularly important for calculating the BBB permeability coefficient.

To evaluate the reliability of our expansion of the *httk* model to include these compartments, we compared its predictions against curated validation datasets using visual predictive checks and quantitative metrics. Despite the limited size of this dataset, the GMFE based on C_max_ values for both the adipose and brain compartments was less than 5. Achieving an error of 2–3 fold is typically the aim of PBPK models in the pharmaceutical industry, where *in vivo* and clinical data are readily available, and Mehta et al. (2024) developed a brain model with minimal *in vivo* data that achieved predictions of C_max_ within 1.25 of experimental levels. However, that publication used a 4-compartment permeability limited brain model with Caco-2 data that were optimized on an individual chemical basis, for five pharmaceutical compounds known to be subject to only passive permeability. Such an approach was not feasible for our generic PBTK model. The fold error of 5 we achieved can be considered a reasonable benchmark for environmental chemicals, where key pharmacokinetic data is often lacking. This was true for predictions from the brain compartment both with and without PFAS. On an individual chemical basis, the majority of data also fell within 5-fold error. Moreover, chemicals were generally over-, rather than under-, predicted, providing a more conservative metric, which would typically be preferred for the risk assessment arena. Such overprediction is commonly seen in high-throughput approaches (Geci et al., 2024; Spaenig et al., 2026).

To better understand some of the limitations of our model, we evaluated chemicals that exceeded a 10-fold difference between experimental and predicted C_max_ values. Weak performance could be explained in several of these instances either by physicochemical properties of the specific chemical or by potential quality issues with the collected *in vivo* experimental data.

- Ambroxol showed a GMFE of 14.0. This high degree of error is perhaps not surprising as ambroxol is a botanical extract, posing challenges for its purity, stability, and overall analysis (Sasidharan et al., 2011; Shipkowski et al., 2018).
- The large error for both dieldrin brain concentrations and deltamethrin adipose concentrations might be due to the lipophilic nature of these chemicals, both with a logP exceeding 5, which may lead to inaccurate predictions through either the calculation of partition coefficients or *in vitro* measurements (Proenca et al., 2021; Geci et al., 2024). For Dieldrin, the large error also calls into question the accuracy of the httk provided intrinsic clearance value of 0 for humans, as rat was not available.
- For two of the chemicals, the elevated fold error may be related to the quality of the analytical data. In reviewing the original raw data, we found that the chromatograms for cinnamic acid and L-methylephedrine showed a low signal-to-noise ratio, which may have led to a larger error in the accuracy of the experimental data.
- The large deviation between experimental and predicted C_max_ values for perfluorooctanoic acid is consistent with findings that necessitated the development of a separate specialized *httk* PFAS model (Crizer et al., 2024). Significant prediction errors for PFAS likely stem from the model’s current inability to account for their strong, saturable plasma protein binding and active, carrier-mediated renal transport, which fundamentally govern their long half-lives and unique distribution dynamics (Ryu et al., 2024; Lin et al., 2023).

The large fold error for the aforementioned chemicals may point to applicability domain limitations of our model. Indeed, flags for PFAS are available in the latest public releases of *httk* as they can be considered outside the applicability domain, and these newer versions offer additional modifications for PFAS (Wambaugh et al., 2026). This is also why we have provided estimates of model performance both with (Figure 5) and without PFAS (Supplementary Figure S9). Transporter substrates is also considered outside the applicability domain of our model, as our perfusion-based BBB does not incorporate active transport mechanisms. Our current framework assumes chemical movement dictated solely by either perfusion-limited or BBB permeability, which is an essential simplification that maximizes high-throughput utility but omits the role of active transport. Transporters like P-glycoprotein and BCRP actively limit brain exposure for many chemicals, potentially lowering the brain concentration below what is predicted by passive diffusion and partitioning alone (Schinkel, 1999; Miller et al., 2008; Agarwal et al., 2011). To focus the model’s assessment on passively driven kinetics, chemicals identified by Yamamoto et al. (2017) as being active transporter substrates using transporter inhibitors (e.g., paliperidone, risperidone) were excluded from model validation. The neglected role of active efflux transporters at the BBB in our model could lead to an overestimation of the parent chemical’s systemic exposure. While we did exclude transporter substrates based on the study by Yamamoto et al. (2017) it is possible that some other chemicals from our test set are also transporter substrates, causing them to likely be overpredicted. Chemicals with a high logP may also be considered outside the applicability domain of our model. The Schmitt method is known to perform more poorly for such lipophilic chemicals (Pearce R. et al., 2017), and lipophilic chemicals are known to be difficult to predict accurately more broadly (Poulin and Haddad, 2012). While these features point to potential applicability domain limitations of our model, due to the lack of data, we could not definitively define an applicability domain in this work.

Indeed, the collected validation dataset is relatively small, limiting major conclusions about model performance. Repeated observations for the same chemical, or from the same study, are found in our datasets. While either of these may inflate model performance, repeated observations from the same chemical may actually highlight the variability in and quality of the underlying *in vivo* data, as different data sources for several chemicals, such as bisphenol A, showed highly variable performance between the experimental and predicted. Sparse datasets limiting interpretation of model performance are an unfortunate reality of working with environmental chemicals, particularly for human tissues from which samples are exceedingly rare. Human C_max_ values used in this work were derived from lumbar cerebrospinal fluid (CSF) rather than brain tissue, and we note that CSF does not necessarily reflect total brain concentrations. We do acknowledge that using CSF as a surrogate for brain tissue introduces an intra-species extrapolation step and that more detailed multi-compartment brain models (Gaohua et al., 2016) would be better suited to the use of CSF data in model evaluation.

As direct human brain-tissue measurements for environmental toxicants are not ethically obtainable, CSF represents the only practical data source for human evaluation; therefore, assessment of the brain compartment is supported primarily by rat data. It was somewhat surprising to us that we found significantly more data for brain than adipose tissue. We acknowledge that a set of only eight chemicals for evaluation of model predictions for adipose is sparse, limiting larger conclusions on the performance of our model. We note that there are additional limitations specific to the adipose compartment. Processes such as lipolysis, weight loss, and exercise can alter adipose concentrations through dynamic mobilization, but these time‐dependent adipose turnover mechanisms are not represented in our current high‐throughput model and therefore constitute an important limitation. Because the adipose compartment is represented using a perfusion‐limited structure without mechanisms for slow lipid turnover or mobilization, the model may underestimate terminal elimination phases for highly persistent, lipophilic chemicals whose clearance depends on gradual release from adipose tissue. Future refinements incorporating dynamic adipose mobilization could improve predictions for chemical classes where this process is known to be significant.

 Additional data for model evaluation would certainly be desirable. However, the goal here was to provide an initial model that expands upon *httk*’s capabilities as a generic high-throughput model. More diverse datasets would require additional animal studies, which come with significant costs, and may poorly reflect human biology for tissues such as the brain (Fritsche et al., 2018; Miller et al., 2020; Molnar et al., 2019; Pollen et al., 2019). Expansion of repositories with highly curated pharmacokinetic data, such as the EPA’s CvT database, would help to better evaluate model performance and refine our model.

Some uncertainties may also be related to the PAMPA data used in this model. The reliability of the predicted brain concentrations is inherently dependent on the applicability of the underlying PAMPA-BBB measurements used in the model. Any experimental variability or systematic bias in these *in vitro* data will propagate into the predicted values and, ultimately, into estimates of brain exposure. Despite the uncertainty associated with available P_e_ estimates, the model structure remains biologically consistent by explicitly representing passive transport across the BBB. Moreover, the *httk* implementation is not restricted to the P_e_ values used in this study and can readily incorporate user-supplied estimates from any experimental or computational source, allowing the model to be updated as more reliable permeability data become available. Another challenge is accurately predicting the partition coefficient (Kp) for highly lipophilic chemicals, particularly difficult with the lipophilic nature of both adipose and brain tissues (Poulin and Haddad, 2012). Although the predicted adipose log C_max_ values strongly correlated with observed data (R^2^ = 0.96), predictions for only five of the eight chemicals fell within a 5-fold error range. Given the small adipose validation dataset, this suggests that while the model captures the linear relationship, it may exhibit systematic bias, likely requiring refined Kp values or further experimental validation. Our model does not incorporate adipose-specific Kp prediction approaches that might improve accuracy, such as using vegetable:oil partition coefficient methods (Poulin et al., 2001; Poulin and Haddad, 2012), as the goal of our generic PBTK model is to aim to be broadly applicable, relying on methods that can be rapidly measured. However, the empirical tissue-specific calibrations of Pearce R. et al. (2017) are applied to adipose based on adipose tissue partitioning data for 75 chemicals. Similar calibrations of the Schmitt (2008) method are applied to each tissue, based on datasets ranging from 39 to 105 chemicals per tissues (Pearce R. et al., 2017).

A limitation that deserves explicit discussion is that the adipose and brain compartments rely on chemical-specific parameters that are not easily measured. Specifically, the adipose tissue partition coefficient (K_adipose2pu_) and the blood-brain barrier permeability parameter (K_pbbb_) are derived from *in vitro* assays or *in silico* predictions. Although these approaches enable high-throughput parameterization across large chemical libraries, they may not fully capture the complexity of the *in vivo* environment. Consequently, uncertainty or bias in K_adipose2pu_ or K_pbbb_ will propagate into predictions of adipose and brain concentrations. As additional high-quality *in vivo* datasets become available, recalibration and validation of these parameters should further improve the predictive accuracy and extend the applicability of the model.

Despite the limited dataset, the reasonable concordance between predicted and observed concentrations supports the utility of these models, particularly in the chemical risk assessment arena. Notably, the diffusion-limited brain compartment model was applied in a recent publication for a tailored IVIVE approach for developmental neurotoxicity, demonstrating its utility in a risk assessment framework (Kreutz et al., 2026). To further enhance the utility of the model, there are numerous future directions this work could be extended.

- Integrating transporter kinetics at the BBB and addressing species-level differences in BBB functional physiology (e.g., transporter density/activity) are key to improving predictive accuracy and RMSE values. Progress is currently limited by the lack of sufficient experimental *in vivo* data for validation with larger, more diverse datasets.
- The brain compartment could be expanded beyond a single brain compartment and single BBB to include the entire central nervous system by sub-compartmentalizing the brain into specific regions and distinct fluid spaces (e.g., cerebrospinal fluid, extracellular fluid) to capture non-uniform distribution. This would also allow better modeling of central nervous system compartments with less-restrictive BBB such as the hypothalamus.
- Our model simulations were conducted using point estimates for all chemical-specific and physiological parameters. We did not perform a formal assessment of uncertainty or variability in these inputs, as the primary objective of the current study was to evaluate baseline model performance under fixed parameter conditions. We also did not perform a parameter sensitivity analysis, as *httk* does not currently provide a built-in sensitivity analysis function. Conducting a comprehensive sensitivity analysis would therefore require development and implementation of an additional analysis workflow using external tools. We acknowledge that the absence of both an uncertainty analysis and a sensitivity analysis represents a limitation of the current work. Future work will therefore include these analyses to evaluate the impact of input parameters on model predictions and to identify key sources of variability and uncertainty.
- It is also important to note that our modified version of *httk* was built on an older release (*httk* v2.2.2). A more recent version of *httk* has since been released. Incorporating the brain and adipose compartments into the latest version of the *httk* package could enhance predictions by leveraging recent improvements to the core framework.

The model we have provided here builds upon the field of high-throughput toxicokinetic modeling. Our model expands upon the existing capabilities of *httk* by providing estimates of adipose and brain concentrations, helping to better assess the kinetics and risks of chemicals, and aiding in prediction of organ toxicity, bioaccumulation potential, or dose optimization for drug development. This generic model fits into the field of high-throughput toxicokinetics with the goal of supporting more rapid, cost-effective screening of chemicals and reducing the need for animal testing. While such generic models may not be as accurate as traditional, chemical-specific models, they provide a base model that can be further refined depending on the context of use. Our model further contributes to the broader next-generation risk assessment framework (Dent et al., 2018; Thomas et al., 2019) of incorporating robust computational methods into regulatory and research applications.

## Data Availability

The original contributions presented in the study are included in the article/Supplementary Material, further inquiries can be directed to the corresponding authors.

## Author disclaimer

The contributions of the National Institutes of Health (NIH) authors are considered Works of the United States Government. The findings and conclusions presented in this paper are those of the authors and do not necessarily reflect the views of the NIH or the U.S. Department of Health and Human Services.
