# Supplementary material for: Extending the high-throughput toxicokinetics R package to include brain and adipose compartments
Source: Front Pharmacol. 2026 Oct 28;17:1895108. doi: 10.3389/fphar.2026.1895108 (PMC13752131; doi:10.3389/fphar.2026.1895108)
Supplement: Supplementary file 6 [file Image5.pdf]

Supplementary Material

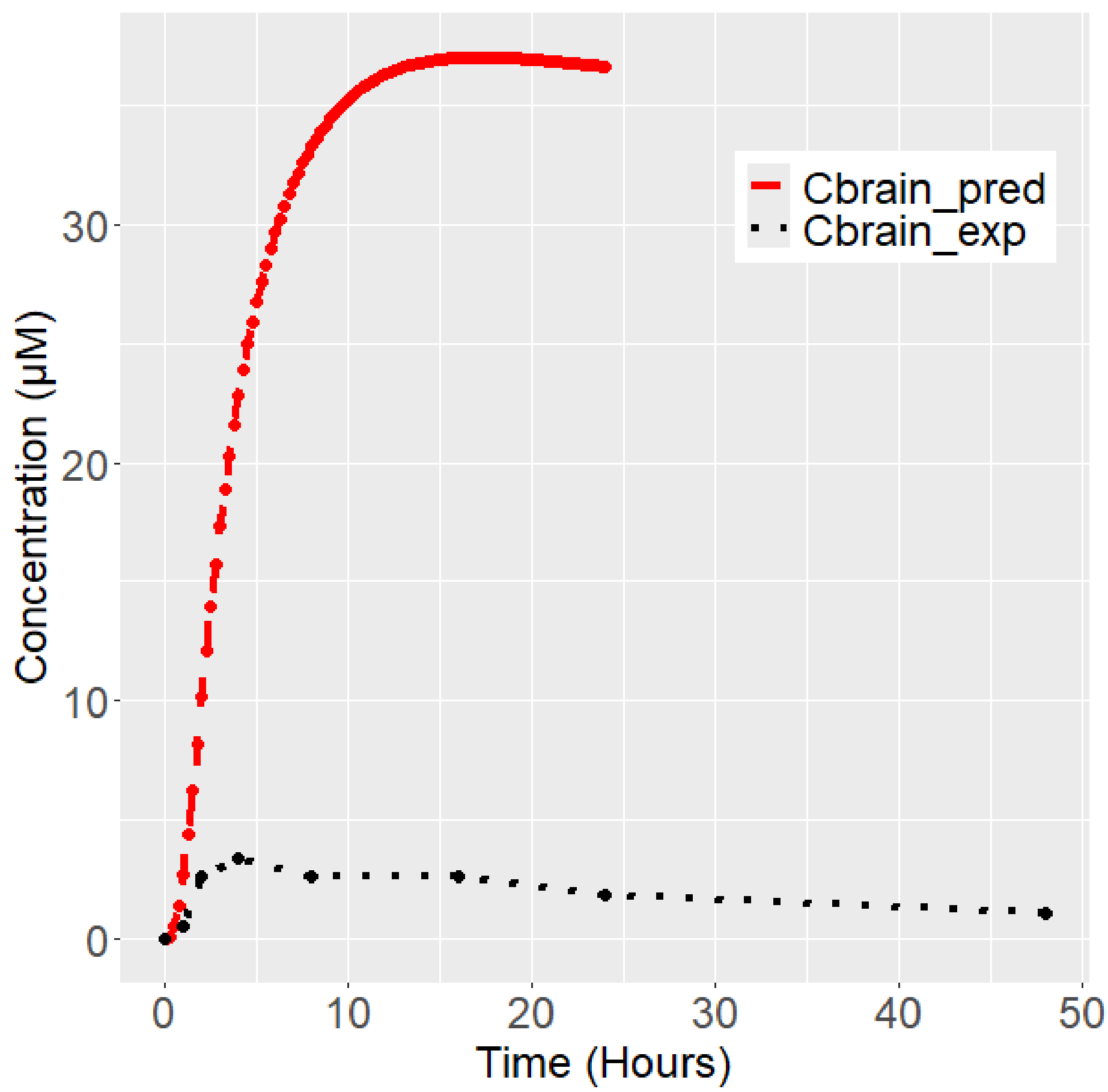

Supplementary Figure 5. Dieldrin experimental data for brain tissue (dotted black line) compared to modified *httk* predictions from the diffusion-limited brain compartment model, with a K_{pbbb} = 0.2191, daily dose = 10 mg/kg, days = 1, doses per day = 1, species = rat, route = oral.
