# Supplementary material for: Extending the high-throughput toxicokinetics R package to include brain and adipose compartments
Source: Front Pharmacol. 2026 Oct 28;17:1895108. doi: 10.3389/fphar.2026.1895108 (PMC13752131; doi:10.3389/fphar.2026.1895108)
Supplement: Supplementary file 10 [file Image9.pdf]

Supplementary Material

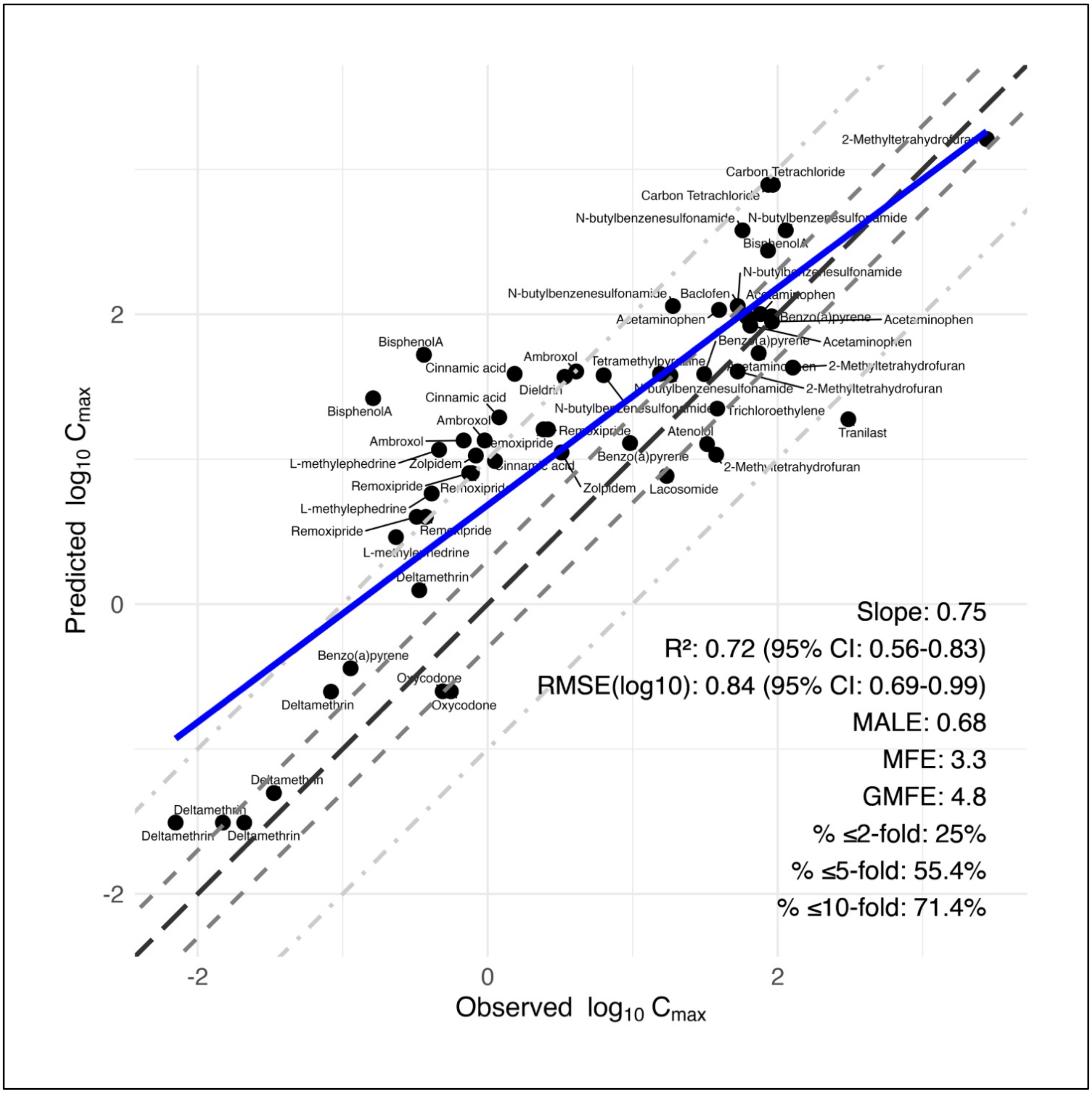

Supplementary Figure 9. Predicted versus observed log C_{max} values in the brain compartment, across 20 chemicals and 56 dosing regimens for the respective species, using the diffusion-limited brain compartment model with PFAS excluded. Dark grey long-dashed line is the line of identity, blue solid line represents the regression line, grey short-dashed lines show ±2-fold, and the grey dot-dashed lines show ±10-fold.
